# Editorial: Advances in proctology and colorectal surgery, volume II

**DOI:** 10.3389/fsurg.2026.1892048

**Published:** 2026-06-24

**Authors:** Marta Goglia, Vito D'Andrea, Mario Trompetto, Giuseppe Clerico, Alberto Realis Luc, Gaetano Gallo

**Affiliations:** 1 Department of Medical-Surgical Science and Translational Medicine, Sapienza University of Rome, Rome, Italy; 2 Department of Surgery, Sapienza University of Rome, Rome, Italy; 3 Department of Colorectal Surgery S. Rita Clinic, Vercelli, Italy; 4 IRCCS Humanitas Research Hospital, Division of General and Digestive Surgery, Milan, Italy; 5 Colorectal Surgery Unit, IRCCS San Raffaele Scientific Institute, Vita-Salute University, Milan, Italy

**Keywords:** artificial intelligence in surgery, colorectal surgery, innovation, minimally invasive techniques, proctology

Colorectal surgery and proctology are undergoing a profound transformation driven by technological innovation, multidisciplinary integration, and increasing attention to patient-centred outcomes (1). The progressive adoption of minimally invasive and robotic techniques, advanced imaging modalities, fluorescence-guided surgery, enhanced recovery pathways, and precision oncology strategies is continuously reshaping the management of both benign and malignant colorectal diseases (2, 3). Beyond technical refinement alone, modern colorectal care increasingly relies on the integration of biological characterization, perioperative optimization, functional preservation, and individualized treatment planning.

At the same time, colorectal surgeons are facing growing clinical complexity. Aging populations, frailty, multimodal oncologic treatments, rare anatomical variants, and the expanding role of immunotherapy and artificial intelligence (AI) are progressively challenging traditional diagnostic and therapeutic paradigms. Consequently, contemporary colorectal surgery is evolving from a predominantly procedure-oriented discipline into a highly personalized and multidisciplinary field requiring close collaboration among surgeons, oncologists, radiologists, gastroenterologists, pathologists, and data scientists.

The studies included in this Research Topic provide a comprehensive overview of several emerging aspects of modern proctology and colorectal surgery, spanning preoperative assessment, intraoperative innovation, postoperative management, predictive oncology, and challenging clinical scenarios. Collectively, these contributions highlight the ongoing transition toward precision-based colorectal care, where technological advances, multidisciplinary decision-making, and individualized patient management are becoming increasingly interconnected in the interest of safer surgery and improved oncologic and functional outcomes.

The preoperative phase in colorectal surgery has advanced into a multidimensional process integrating oncologic staging, physiological optimization, and individualized treatment planning. Several scientific contributions highlight the growing role of advanced imaging, predictive models, and tailored perioperative strategies in improving surgical safety and oncologic outcomes. Chai et al. explored the value of MRI-based biomarkers in rectal adenocarcinoma, demonstrating how amide proton transfer (APT) imaging and T1 mapping may help identify aggressive pathological features and support personalized treatment stratification. The correlation between imaging parameters and Ki-67 expression further supports the growing relevance of functional imaging as a tool for preoperative biological characterization and personalized therapeutic stratification. These findings further reinforce the emerging role of radiomics and quantitative imaging in preoperative colorectal cancer assessment. The importance of perioperative risk prediction was highlighted by Yu et al. which identified several predictors of postoperative haemorrhage after proctologic surgery in diabetic patients. Male sex, hypertension, preoperative antibiotic administration, and postoperative constipation were identified as significant risk factors associated with haemorrhagic complications. Although focused on postoperative complications, the study underlines the importance of accurate preoperative identification of vulnerable patients and multidisciplinary perioperative management. Moreover, preoperative care of unplanned emergency presenting colorectal malignant lesions such as colorectal obstruction was addressed by Huang et al. who proposed a low-cost decompressive strategy for acute left-sided colonic obstruction. The authors highlighted how simplified bridge-to-surgery approaches may represent valuable alternatives in highly selected resource-limited settings.

Preoperative complexity was further illustrated by Xia et al. describing a rare case of paraneoplastic myasthenia gravis during neoadjuvant immunotherapy for rectal cancer. Beyond its rarity, the report reflects the increasing complexity of multidisciplinary decision-making in the era of modern oncologic and immunotherapeutic treatments.

Furthermore, increasing number of geriatric population is an important aspect of modern management of colorectal surgical plans.

The study of Montroni et al. emphasized how advanced imaging, multidisciplinary evaluation, and emerging AI tools may support frailty based assessment and personalized neoadjuvant strategies while balancing oncologic efficacy and treatment-related toxicity in elderly affected patients (4).

Even technical intraoperative advancements are continuously emerging and the surgery is increasingly characterized by the integration of precision technologies aimed at improving safety while preserving oncologic and functional outcomes (5–7).

Litchinco et al. highlighted the growing role of indocyanine green fluorescence imaging in preventing iatrogenic ureteral injuries during complex pelvic surgery. Across the included studies, visualization rates consistently exceeded 95%, particularly in minimally invasive and robotic procedures, supporting the value of fluorescence guidance in anatomically challenging settings. Importantly, the review also discussed emerging renally excreted fluorophores capable of intravenous administration, potentially overcoming the limitations associated with ureteral catheterization and further simplifying operative workflow. Indeed, accordingly to current literature the review reflects the broader evolution toward image-guided and augmented intraoperative navigation in minimally invasive and robotic colorectal procedures (8, 9). The aim of reduced surgical invasiveness was also explored by Malev et al. who demonstrated favourable perioperative and oncologic outcomes following natural orifice specimen extraction surgery (NOSES). The study demonstrated that NOSES was associated with lower intraoperative blood loss, faster recovery of gastrointestinal function, shorter hospital stay, and a lower overall complication rate, without compromising oncologic adequacy or long-term survival outcomes. These findings support the ongoing transition toward scar-reducing and function-preserving surgical approaches.

However, postoperative care remains a key determinant of recovery and long-term oncologic outcomes following colorectal surgery. Contemporary management increasingly focuses on inflammatory modulation, functional rehabilitation, and precision surveillance. The scoping review by Alves Bersot et al. demonstrated that Enhanced Recovery After Surgery (ERAS) protocols were consistently associated with lower postoperative levels of inflammatory biomarkers such as C-reactive protein (CRP) and interleukin-6 (IL-6), together with reduced leukocyte response and improved recovery parameters, reinforcing the importance of multimodal perioperative care in improving recovery and potentially modulating oncologic outcomes.

Functional postoperative complications were explored in the paper by Cao et al., which highlighted the diagnostic challenges of occult anastomotic dysfunction after low anterior resection. The report highlighted how conventional diagnostic criteria based exclusively on luminal caliber or endoscopic passage may fail to identify functional abnormalities of the anastomosis and the need for integration of patient-reported symptoms and functional assessment into postoperative evaluation. Another hot topic in colorectal surgery is the postoperative surveillance. Quanwei et al. reported a rare delayed recurrence of rectal mixed adenoneuroendocrine carcinoma more than seven years after surgery (10). They underlined how molecular and immunohistochemical characteristics, including Ki-67 expression and neuroendocrine markers, may influence postoperative prognosis and metastatic potential.

Several additional contributions addressed broader aspects of colorectal disease management, including predictive oncology, surveillance strategies, epidemiologic controversies, and advanced metastatic therapies. Zeng et al. proposed a prognostic nomogram capable of improving risk stratification beyond conventional purely anatomic TNM staging alone, reflecting the growing role of integrated predictive modeling in colorectal oncology.

Similarly, He et al. emphasized the importance of tailored surveillance strategies and molecular profiling in identifying patients at increased risk of synchronous premalignant lesions. Moreover, the study emphasized how incomplete preoperative colonoscopy, frequently encountered in obstructive colorectal cancer or inadequate bowel preparation, may contribute to missed lesions and subsequent metachronous colorectal cancer.

The growing application of advanced epidemiologic methodologies was illustrated by Wei et al. who used Mendelian randomization analysis to demonstrate the absence of a causal association between appendectomy and colorectal cancer risk.

A multimodal salvage therapy for microsatellite-stable colorectal liver metastases was reported by Zheng et al., who addressed the challenging scenario of microsatellite-stable colorectal cancer liver metastases (MSS-CRCLM) after failure of multiple lines of systemic therapy. The retrospective study demonstrated encouraging oncologic outcomes using a combination of hepatic arterial infusion chemotherapy (HAIC), anti-angiogenic therapy with fruquintinib, and immunotherapy with tislelizumab (11). The reported objective response rate of 42.2% and median overall survival exceeding 15 months appear particularly relevant considering the historically poor prognosis of heavily pretreated MSS metastatic colorectal cancer. The authors highlighted ongoing efforts to overcome immunoresistance in advanced colorectal cancer.

Several rare clinical scenarios highlighted the importance of individualized surgical planning and advanced anatomical understanding in colorectal surgery.

Chen et al. provided one of the largest contemporary series dedicated to presacral tumours, a rare and embryologically heterogeneous group of lesions arising within the retrorectal space. The study emphasized the substantial diagnostic complexity associated with these tumors, reporting a misdiagnosis rate approaching 30%, most commonly due to confusion with benign anorectal conditions such as fistulas, abscesses, or pilonidal disease (12).

They underlined the diagnostic complexity and multidisciplinary management required for rare retrorectal tumors, while also demonstrating favorable outcomes following specialized surgical treatment.

An equally challenging anatomical scenario was described by Su et al. who illustrated how minimally invasive colorectal surgery can be safely performed even in patients with mirror-image anatomy through meticulous preoperative planning and adaptation of surgical ergonomics and vascular dissection strategies.

Overall, the studies included in this Research Topic highlight the ongoing evolution of colorectal surgery toward increasingly personalized, technology-driven, and multidisciplinary care. Continued integration of advanced imaging, precision surgery, perioperative optimization, and biologically informed strategies will likely further improve both oncologic and functional outcomes in colorectal disease management.
